# Significance of incident reports by medical doctors for organizational transparency and driving forces for patient safety

**DOI:** 10.1186/s13037-020-00240-y

**Published:** 2020-04-16

**Authors:** Tatsuya Fukami, Masakazu Uemura, Yoshimasa Nagao

**Affiliations:** Department of Patient Safety, Nagoya University Hospital, Nagoya, Japan

## Abstract

**Background:**

Incident reporting is an effective strategy used to enhance patient safety and quality improvement in healthcare. An incident is an event that could eventually result in harm to a patient. The aim of this study is to re-evaluate the importance of reporting by medical doctors to improve quality in healthcare and patient safety.

**Methods:**

We conducted a retrospective analysis of the reported incidents registered in our institutional database from April 1st 2015 to March 31st 2019, classified according to eight variables proposed by the National University Hospital Council of Japan, to determine the type of incidents and their potential harm to patients.

**Results:**

Registered reports totalled 43,775, approximately 8% of which arise annually from medical doctors in clinical departments. Incidents with higher impact on patients have significantly increased the rate of reporting by medical doctors. The most frequent types of report overall concerned medication incidents, followed by infusion lines, drainage-tube devices, cure, examination, and treatment outside the operating room. The most frequent reports by medical doctors involved operation-related incidents, followed by cure, examination, treatment outside the operation room, and medications.

**Conclusion:**

Reporting by medical doctors reflects the organizational transparency and the driving forces behind patient safety and quality improvement in healthcare. Efforts toward seamless improvement in patient safety and quality at our hospital continue apace.

## Background

A reporting culture means cultivation of the atmosphere whereby workers in a hospital are able to report patient safety concerns with fairness and without fear of blame [1]. Confidentiality will be guaranteed and the reports submitted by the employee will be acted upon for improvement. Moreover, the report will be recognized as a worthy act. An incident-reporting system is an organized approach to reporting near misses or adverse events to enable improvement [2]. An incident-reporting system is a voluntary, anonymous, confidential electronic system that allows the reporting of incidents and adverse events for analysis by experts in quality improvement and patient safety [3–5]. Although this system might well improve patient safety by reducing the risk of adverse events, it has many obvious limitations: reports are sometimes entirely subjective and therefore unfair, are not comparable between hospitals, and carry unacknowledged bias [6, 7].

This is a result of a third party reviewing medical records at hospitals nationwide and picking up adverse events according to certain criteria. Figure 2 shows the estimates based on the results of this study and the numbers reported by our hospital last year.

The purpose of this study is to re-evaluate the importance and submitting activity of incident reports by medical doctors to grasp the critical events in hospital and ensure organizational transparency.

## Methods

Our centre is a 1000-bed academic hospital with a clinical patient safety and quality management department that oversees patient safety affairs and is responsible for the incident-reporting system. Patient safety incident reporting is mandatory for all staff in our hospital, including contracted workers, when they confront an incident. We surveyed the incident reports submitted from April 1st 2015 to March 31st 2019, selected from all incidents reported by all hospital workers, and collected information from the corresponding original electronic incident reports. We compared the reports by medical doctors and other workers in the hospital. The collected data included: the date of the incident; ward/department where the incident occurred; healthcare profession, years of experience, and affiliated department of the reporter and person involved in the incident; information regarding the patient; incident details; incident classification; and incident severity classification. Incident severity and continuity classification is widely used in Japanese hospitals to evaluate the impact on the patient conveyed by the incident, and is based on a classification system developed by the National University Hospital Council of Japan [8]. The system classifies eight ranks by incident severity and continuity impact for patients (Table 1). Levels from 0 to 1 are defined as near miss, whereby an unexpected event has the potential to cause, but does not actually harm the patient or interrupt the normal situation. A near miss is often an error prevented by other circumstances. Levels from 2 to 5 are defined as adverse event, which represents any unintentional or unfavourable clinical sign or symptom, including complications, any new illness or disease or the deterioration of existing disease or illness, and any clinically significant deterioration in any laboratory assessments or clinical tests. The most commonly used tool in quality monitoring is the Pareto chart. In this chart, the values are presented in decreasing order and the cumulative function is a concave shape. A Pareto chart is deemed appropriate for the first three steps in the problem-solving process (i.e., clarifying and breaking down the problem, then setting the target) because a Pareto chart aims to highlight the most important causal factors. The electronic incident reporting system used by our hospital is Incident Report System version 1.0 (Safe Master, Fukuoka, Japan). We extracted only necessary incident information items for this study, and processed information concerning individuals (e.g., the reporter and target patient) anonymously. People who notice the event will report. It can be reported by multiple people in the same occupation, by multiple occupations, or by only one person. All analyses were performed using SPSS version 25 (IBM, Armonk, NY, USA). About patient and public involvement statement, this study was approved by the Institutional Review Board of the study hospital.

**Table 1:** Incident severity classification system recommended by the National University Hospital Council of Japan

Level	Continuity of injury	Severity of injury	Outcome/Treatment of injury
Level 0	–	–	Error or trouble with a pharmaceutical or medical device was found, but did not affect the patient
Level 1	None	–	There was no harm to the patient (but there was a possibility of some influence)
Level 2	Transient	Mild	Treatment was not necessary (mild change in vital signs, need for increased patient observation, examination for confirmation of safety, etc.)
Level 3a	Transient	Moderate	Simple treatment was required (disinfection, poultice, skin suture, administration of analgesics, etc.)
Level 3b	Transient	Severe	Substantial treatment was required (significant change in vital signs, use of artificial respirator, surgery, prolongation of hospitalization, hospitalization, fracture, etc.)
Level 4a	Permanent	Mild to moderate	Permanent disability or subsequent complication remained, but was not accompanied by significant dysfunction or an aesthetic problem
Level 4b	Permanent	Moderate to severe	Permanent disability or subsequent complication remained, accompanied with significant dysfunction or an aesthetic problem
Level 5	Death	–	Death (excluding those due to the natural course of the underlying disease)

## Results

**Fig. 1:**
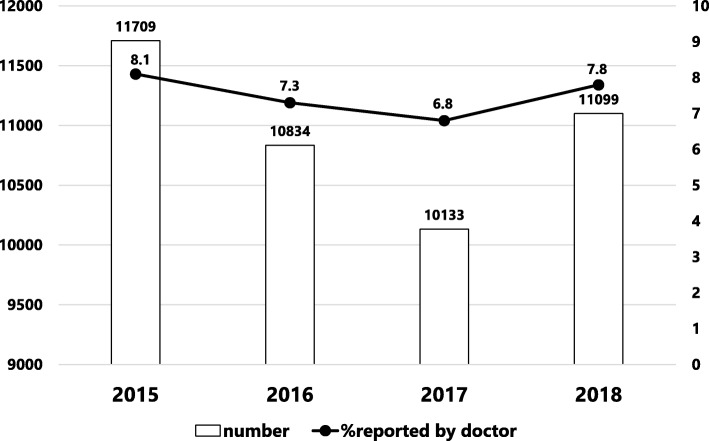
Total number of incident reports and percentage reported by medical doctors in each fiscal year

**Fig. 2:**
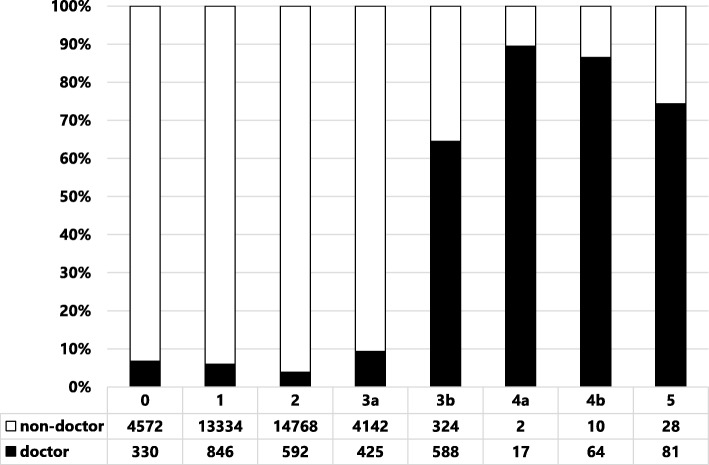
Distribution of incidents reported by medical doctors and non-medical doctors

When patient safety incidents and accidents occur in the hospital, they are submitted to general risk and safety managers from various occupations via an electronic reporting system; over 10,000 cases are reported each year, and the total number of incident reports hospital-wide is 43,775 (Fig. 1). A review of the incidents reported by medical doctors each year revealed that approximately 8% arose from all clinical departments (Fig. 1). The incidents with higher impact on patients significantly increased the number of reports by medical doctors (Fig. 2).

**Fig. 3:**
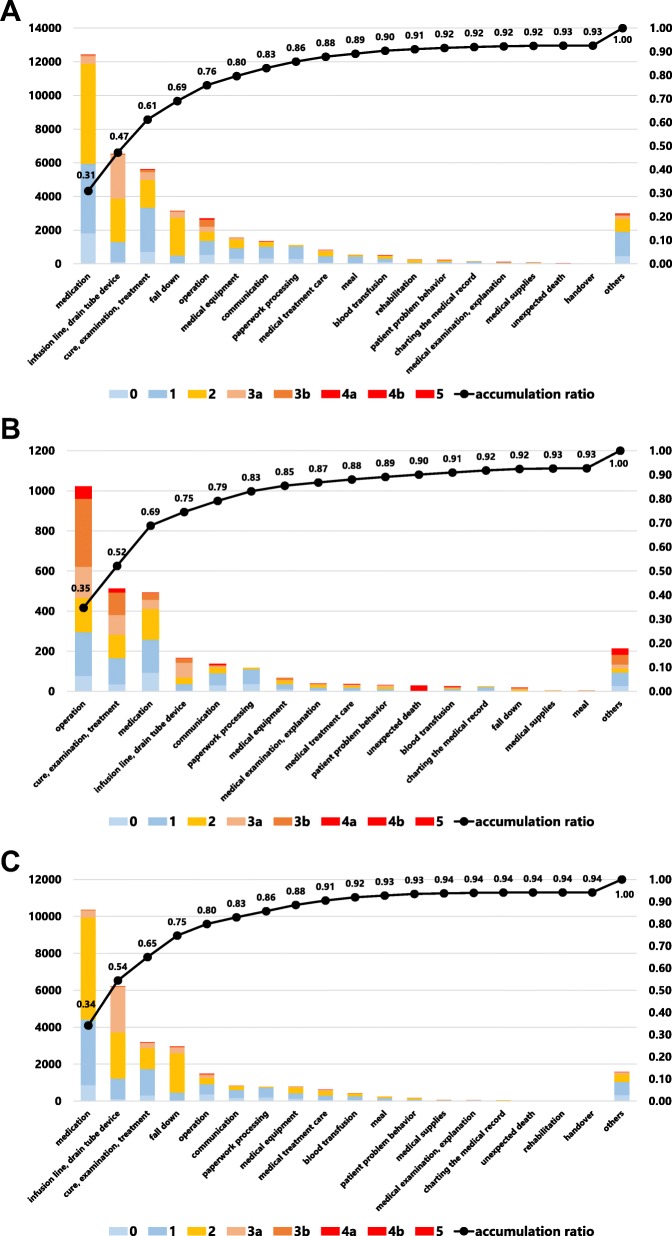
Pareto chart showing incident-reporting frequency and severity for each event: (**a**) overall, (**b**) medical doctors, (**c**) nurses

We classified incident reports using a Pareto chart. The incident reports from nurses totalled 30,392, from doctors 2943, and from others 6488 for this 4-year analysis. The most frequent type of report was medication incidents, followed by infusion lines and drainage-tube devices, and cure, examination and treatment outside the operating room (Fig. 3a). The most frequent reports by medical doctors concerned operation-related incidents, followed by cure, examination and treatment outside the operating room, and medications (Fig. 3b). Nurses most frequently reported incidents related to medication, followed by infusion lines and drainage-tube devices, and cure, examination and treatment outside the operating room (Fig. 3c).

**Fig. 4:**
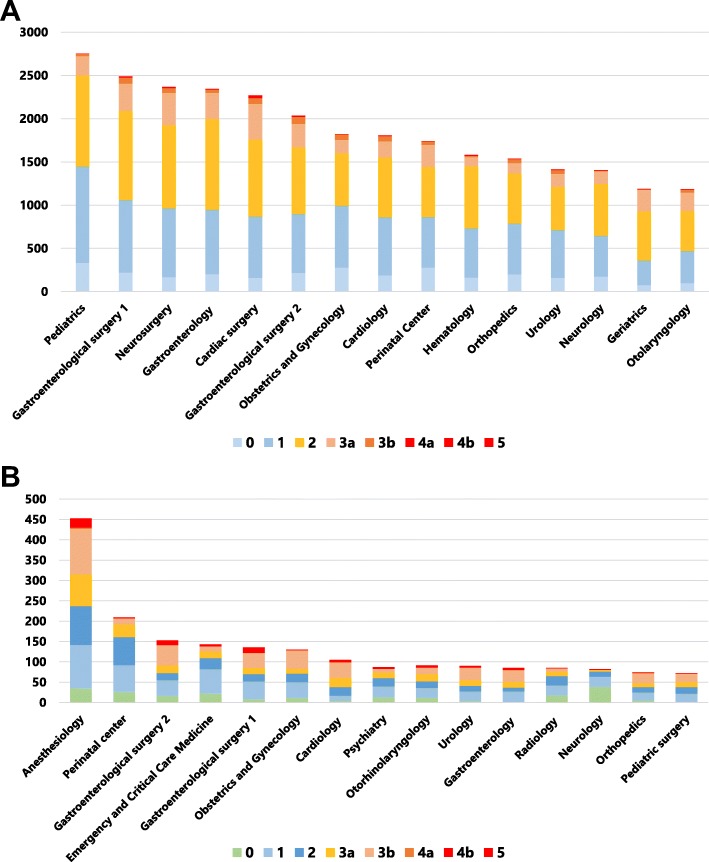
Pareto chart showing incident reporting frequency and severity. **a** Cases reported according to clinical department. **b** Cases reported by doctors according to their clinical department

The clinical department in which the incidents occurred were reported by all workers. The department with the largest number of reports was Paediatrics, followed by Gastroenterological Surgery 1 and Neurosurgery (Fig. 4a). Based on reporting solely by doctors, reports came most frequently from Anaesthesiology, followed by the Perinatal Centre and Emergency and Critical Care Medicine (Fig. 4b).

## Discussion

This study covers identification of the high-risk areas in the hospital and the importance of incident reporting from medical doctors. The most frequently reported departments are not “dangerous departments”, but “accident extraction power and transparency”, which should be highly evaluated.

Although the incident reporting system does not reflect the actual hospital-wide events in our centre, it is a worthwhile source of information from which to discover potential risks and attributable factors of a representative patient safety issue [3, 9]. In this system, events whose potential consequences are difficult to measure in patient prognosis and which have been caused by external events or inappropriate or defective internal processes, systems, and/or systemic improvement activity are also indicated as risks [10]. Accumulation of near-miss incidents of the same type and with a small impact as a one-off event also carries the risk of potential adverse events. There is a positive correlation between the number of incident reports and improved safety culture within the organization [11]. Our hospital is a leading centre in regard to the number of incident reports (Fig. 1) in Japan. Adverse events reported by medical doctors helps to coordinate the treatment of severe and chronic injury and is important for continued transparency and active reporting in the hospital. Certain specialties have reported more near misses than others, and doctors have reported more harm incidents than near misses [11], in line with our present results (Fig. 2). According to another study, events with no harm to the patient represented a high percentage of all incident reports from nurses, other healthcare providers, and other workers [12]. We assessed the various factors involved using a Pareto chart, the purpose of which is to highlight the most important among a large set of factors. The major issue hospital-wide (Fig. 3a) and among nurses (Fig. 3c) is medication errors, while for doctor operation-related matters are of greatest concern (Fig. 3b). Our aim is to target the outstanding issues and consider appropriate countermeasures for each incident, which can be complicated by many factors. The countermeasures should be comprehensive and practical. Furthermore, we investigated which department has risks with or without errors. Paediatrics and surgical departments are major sources of reported incidents (Fig. 4a). Paediatric patients are of varying age and carry a wide disease spectrum that can easily deteriorate. Healthcare providers should thus customize treatment for each patient with detailed assessment of the individual’s condition. Surgical departments obviously are sensitive to various risks during preoperative diagnosis, surgical procedures, and postoperative treatments. Our reporting standards include the occurrence of complications so that we can pick up on potential risks when there is a recurrent complication. As well as surgeons, anaesthesiologists face many challenging tasks and risks (Fig. 4b). Once again, bold and honourable reporting of incidents drives the safety culture and leads to organizational transparency in anaesthesiology. Overseeing measures to assess the reduction or increase in near misses or adverse events is a useful approach to improve the effectiveness of an incident-reporting system. Incident-reporting analyses demonstrate two influential factors, systemic issues and human errors. To reduce the hospital-wide risk, prompt, correct, and fair incident reporting is mandatory for improvement in healthcare [13, 14].

## Conclusion

In conclusion, reporting of incidents by medical doctors reflects the organizational transparency and the drive toward patient safety and quality improvement in healthcare. In addition, the reporting of near-miss events hospital-wide also assumes importance because they are the sentinel for future adverse events. After identifying the high-risk areas in various clinical departments, the next step should be to analyse the root cause of incidents, especially those reported by doctors, and intervene appropriately to improve the quality of healthcare. This should contribute directly to safer care and the overall drive toward the enforcement of a culture of patient safety in the hospital. We can say that reports from doctors are overwhelmingly more severe than reports from other occupations. This means that hospitals cannot accurately ascertain adverse events unless there are few reports from doctors. As a safety manager, we want to clear adverse events as much as possible, and to respond to particularly serious adverse events by collecting the best of the hospital. Nurses can report many attempted and harmless cases, minor cases such as abrasions and bruises, but that alone is not enough. Our hospital is grasping the overall picture of adverse events due to the increase in reports from doctors, and feels that we can finally stand at the starting point of medical safety.

Reporting by medical doctors reflects the organizational transparency and dynamic efforts required for patient safety and quality improvement in healthcare. Efforts to achieve seamless improvement in patient safety and care at our hospital will continue.

## Data Availability

All data generated or analysed during this study are included in this published article.
